# Dissociated neurovascular dynamics in *ATP1A2*-related familial hemiplegic migraine mimicking acute ischemic stroke

**DOI:** 10.1186/s12883-026-04930-5

**Published:** 2026-05-11

**Authors:** Gha-hyun Lee, Jiyoung Kim, Jae Wook Cho

**Affiliations:** 1 Department of Neurology, Biomedical Research Institute, Pusan National University Hospital, 179 Gudeok-ro, Seo-gu, Busan, 49241 Korea, Republic Of; 2 Pusan National University School of Medicine and Medical Research Institute, Yangsan, Korea, Republic Of; 3 Department of Neurology, Pusan National University Yangsan Hospital, Yangsan, Korea, Republic Of

**Keywords:** Familial hemiplegic migraine, *ATP1A2* mutation, Neurovascular coupling, Cortical spreading depression, Susceptibility-weighted imaging

## Abstract

**Background:**

Hemiplegic migraine (HM) is a rare migraine subtype with motor aura. Familial hemiplegic migraine type 2 (FHM2), caused by *ATP1A2* mutations, often mimics acute stroke, making early neuroimaging differentiation critical. While dynamic perfusion changes are documented during attacks, the dissociated neurovascular dynamics with reduced macrovascular flow signal and presumed microvascular hyperemia has not been well characterized.

**Case presentation:**

A 37-year-old man presented with acute severe headache and global aphasia. Initial diffusion-weighted MRI was unremarkable, but time-of-flight magnetic resonance angiography (TOF-MRA) demonstrated attenuated vascular signal in the left middle and posterior cerebral arteries without fixed stenosis. Concurrently, susceptibility-weighted imaging (SWI) revealed prominent cortical veins in the same territories, indicating a dissociated perfusion pattern. Electroencephalography showed left hemispheric slowing. Symptoms resolved completely within 72 h. Genetic testing confirmed a heterozygous pathogenic mutation (c.1816G > A, p.Ala606Thr) in *ATP1A2*, consistent with FHM2.

**Conclusions:**

This case highlights a unique neurovascular dissociation in FHM2, where macrovascular signal attenuation and presumed microvascular hyperemia coexist during the acute phase. These findings suggest that *ATP1A2*-related astrocytic dysfunction and cortical spreading depression lead to layered, asynchronous vascular dysregulation. Recognizing this reversible pattern is essential to distinguish FHM2 from stroke mimics and prevent inappropriate thrombolytic interventions.

## Background

Familial hemiplegic migraine (FHM) is a rare autosomal dominant subtype of migraine with aura, characterized by transient motor weakness. Among its genetic variants, FHM type 2 (FHM2) is caused by mutations in the *ATP1A2* gene, which encodes the α2 subunit of the Na+/K+-ATPase pump [1, 2]. In the central nervous system, this pump is primarily expressed in astrocytes and plays a crucial role in maintaining ion homeostasis by clearing potassium and glutamate from the synaptic cleft [3]. Consequently, *ATP1A2* mutations lead to increased neuronal excitability and a lower threshold for cortical spreading depression (CSD), the underlying pathophysiological mechanism of the migraine aura [4]. 

Clinically, FHM2 often presents as a formidable stroke mimic, as the sudden onset of hemiparesis and aphasia can be indistinguishable from an acute ischemic stroke. This diagnostic ambiguity poses a significant challenge in emergency settings, where the inappropriate administration of thrombolytic therapy may occur. While dynamic perfusion abnormalities—ranging from hypoperfusion to hyperperfusion—have been documented during FHM attacks, the underlying hemodynamic mechanisms remain complex and heterogenous across different phases of the attack [5–7]. Specifically, the simultaneous coexistence of macrovascular signal attenuation and presumed microvascular hyperemia has been rarely characterized in FHM2. This “dissociated” pattern suggests a layered, asynchronous vascular dysregulation that reflects the unique neurovascular uncoupling associated with astrocytic dysfunction [8–10]. 

In this report, we present a genetically confirmed case of FHM2 featuring this distinct radiological signature on multimodal MRI. By demonstrating the concurrent occurrence of macrovascular signal reduction on time-of-flight magnetic resonance angiography (TOF-MRA) and prominent cortical veins on susceptibility-weighted imaging (SWI), we aim to provide deeper insights into the complex pathophysiology of FHM2 and emphasize the importance of identifying these reversible patterns to prevent misdiagnosis and unnecessary interventions.

## Case presentation

A 37-year-old man presented to the emergency department with acute severe headache and aphasia. The patient was last known well when he complained of headache to his family before they left home. Approximately two hours later, the family checked a home monitoring camera and found him lying on his side unresponsive, prompting emergency services to be called. He was initially evaluated at a local hospital, where non-contrast CT brain showed no acute abnormalities. He was subsequently transferred to our institution, arriving approximately 6 h and 30 min after the last known well time. As this exceeded the 4.5-hour intravenous thrombolysis (IVT) window, IVT was not administered. Upon arrival, his National Institutes of Health Stroke Scale (NIHSS) score was 7 (due to global aphasia), though he remained hemodynamically stable and alert. Initial neurological examination confirmed global aphasia, but no definite focal motor weakness was identified.

Initial laboratory tests including complete blood count, electrolytes, creatine kinase, and coagulation studies were unremarkable. Cerebrospinal fluid analysis revealed normal opening pressure and cerebrospinal fluid composition. Extensive infectious workup, including cerebrospinal fluid next-generation sequencing (NGS) targeting common bacterial, viral, and fungal pathogens, as well as bacterial, mycobacterial, and fungal cultures and tuberculosis PCR, yielded negative results. Electroencephalography (EEG) demonstrated marked left hemispheric slowing without epileptiform discharges.

**Fig. 1:**
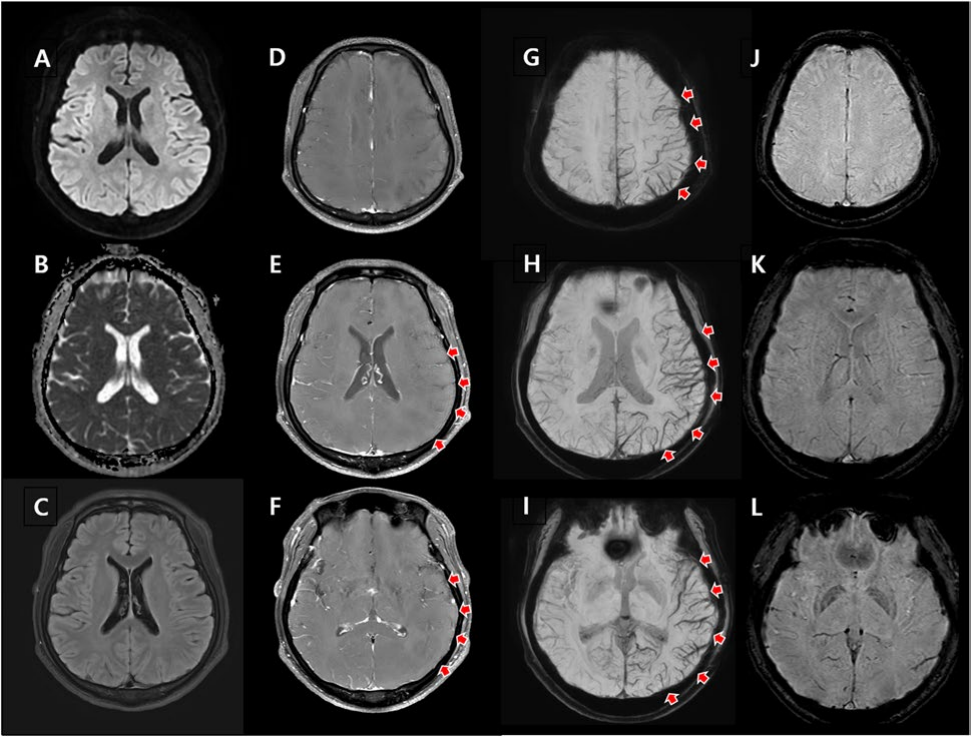
Multimodal brain MRI on the day of headache onset (**A–I**) and at follow-up (**J–L**) in a patient with familial hemiplegic migraine type 2 (FHM2). **A-C** Initial axial DWI, ADC, and FLAIR images (7 h after symptom onset) show no diffusion restriction or parenchymal signal changes. **D–F** Post-contrast T1-weighted images demonstrate decreased enhancement in the left hemisphere compared to the right. (red arrows). **G–I** Susceptibility-weighted imaging (SWI; GE SWAN, MinIP reconstruction) at the time of acute presentation reveals prominent cortical veins in the left hemisphere, suggesting asymmetric microvascular hyperemia (red arrows). **J–L** Follow-up SWI (Siemens, magnitude images) on hospital day 3 demonstrates complete resolution of the previously observed cortical venous prominence

**Fig. 2:**
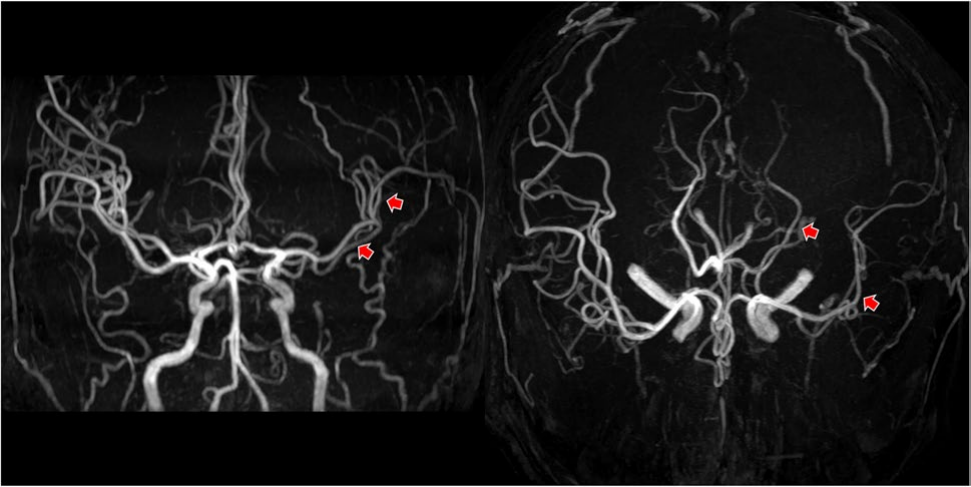
Time-of-flight magnetic resonance angiography (TOF-MRA) findings. TOF-MRA during acute phase (7 h after onset) demonstrates attenuated vascular signals and reduced vascular conspicuity in the left middle cerebral artery and posterior cerebral artery branches compared to the contralateral side, without definite evidence of stenosis. (red arrows)

Brain computed tomography (CT) scan was unremarkable. Seven hours after symptom onset, multimodal MRI was performed. Diffusion-weighted imaging (DWI) showed no evidence of acute infarction, and fluid-attenuated inversion recovery (FLAIR) sequences were normal (Fig. 1A-C). However, TOF-MRA revealed attenuated vascular signals in the left middle and posterior cerebral artery territories without fixed stenosis (Fig. 2). Contrast-enhanced MRA performed showed no evidence of fixed stenosis or occlusion. Post-contrast T1-weighted images also demonstrated relatively decreased parenchymal enhancement in the left hemisphere compared to the right, suggesting a dissociated hemodynamic pattern. (Fig. 1D-F) SWI (GE SWAN MinIP, TR 43.1 ms, TE 25.0 ms, flip angle 15°, MinIP slab 16 mm) showed prominent cortical veins in the left hemisphere (Fig. 1G-I).

The patient’s aphasia resolved completely within 24 h without specific treatment, while right-sided numbness and headache persisted for an additional two days before gradual improvement. After symptom resolution, a detailed history revealed recurrent episodes of headaches accompanied by transient right-sided weakness since adolescence. His father also had a similar history of recurrent headaches with transient limb weakness, suggesting a familial disorder.

**Fig. 3:**
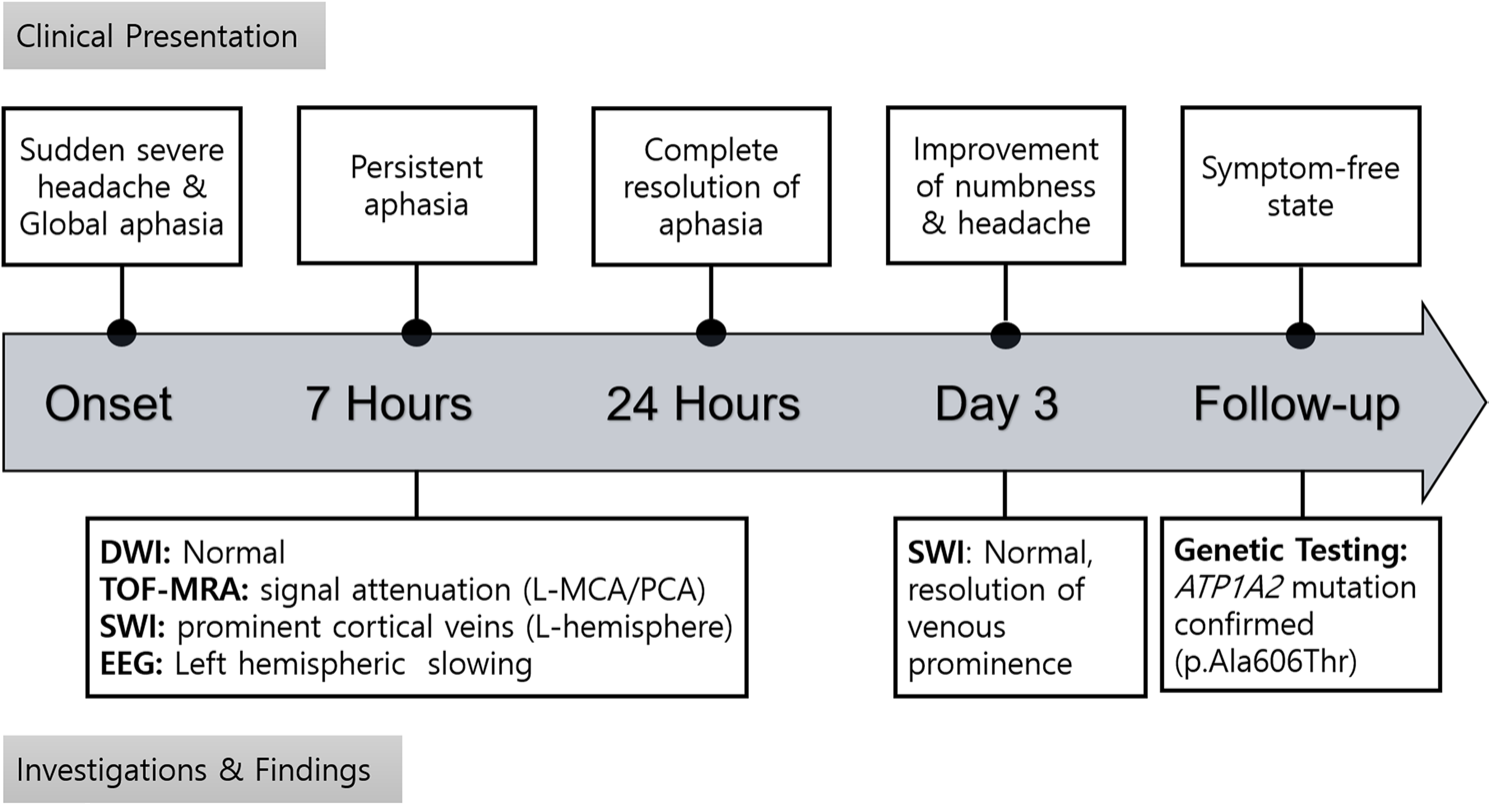
Clinical and diagnostic timeline of the FHM2 case

Given the recurrent stereotyped episodes and positive family history, targeted genetic testing was performed and identified a heterozygous missense mutation in *ATP1A2* (NM_000702.4:c.1816G > A, p.Ala606Thr), which has been previously established as a pathogenic variant for FHM2 [11]. A follow-up SWI (Siemens magnitude, TR 53.0 ms, TE 40.0 ms, flip angle 15°, slice thickness 2.3 mm) on hospital day 3 showed complete resolution of the previously observed cortical venous prominence (Fig. 1J-L). The clinical course and diagnostic timeline are summarized in Fig. 3.

## Discussion

The present case illustrates the diagnostic complexity and an unusual hemodynamic presentation of FHM2, characterized by acute aphasia mimicking ischemic stroke, in the absence of diffusion-restricted lesions or structural abnormalities on conventional MRI. The paradoxical combination of reduced vascular conspicuity on TOF-MRA and prominent cortical veins on SWI underscores the complexity of neurovascular alterations in hemiplegic migraine and poses a significant diagnostic challenge in the acute setting.

FHM2, accounting for approximately 20% of FHM cases is caused by pathogenic variants in *ATP1A2*, encoding the astrocytic Na⁺/K⁺-ATPase α2 subunit, which plays a pivotal role in potassium buffering and glutamate clearance [2, 12]. Dysfunction of this astrocytic pump lowers the threshold for cortical spreading depression (CSD), leading to prolonged aura symptoms and transient neurological deficits [13]. Importantly, astrocytes are integral to neurovascular coupling; [14] therefore, *ATP1A2*-related dysfunction may result not only in neuronal excitability changes but also in abnormal regulation of cerebral blood flow [13–15]. 

In this case, post-contrast T1-weighted images demonstrated decreased parenchymal enhancement, and TOF-MRA showed decreased conspicuity of left-sided cerebral arteries without evidence of fixed stenosis or occlusion. Given the flow-dependent nature of TOF-MRA, these findings are more consistent with transient vasoconstriction or slow blood flow rather than true macrovascular obstruction. - an interpretation further supported by the absence of stenosis or occlusion on concurrent contrast-enhanced MRA [6, 10]. Concurrently, SWI revealed prominent cortical veins in the same hemisphere. The cortical venous prominence observed on SWI most likely reflects an increased local deoxyhemoglobin concentration and/or delayed venous flow, consistent with the established physiological basis of the SWI venous signal [16]. ATP1A2 dysfunction impairs astrocytic clearance of extracellular K⁺ and glutamate, sustaining neuronal excitation and potentially increasing metabolic demand [14]. This metabolic strain, combined with the observed slow arterial flow, may lead to an increased oxygen extraction fraction (OEF) and blood stasis within the venous system. Consequently, the elevated level of paramagnetic deoxyhemoglobin renders the cortical veins more prominent on SWI [16]. Additional mechanisms may also contribute: reduced arterial driving pressure may impair venous drainage, leading to increased venous blood volume; and relative hypoperfusion may result in increased OEF and accumulation of metabolic byproducts [5, 10]. The available qualitative imaging data do not permit definitive distinction between these mechanisms.

The reduced MRA signal in the left MCA territory most likely reflects the oligemic phase of CSD-a well-characterized, transient reduction in cerebral blood flow following the initial cortical depolarization wave-rather than structural vasoconstriction [17]. In this framework, aphasia arises from CSD-mediated suppression of neuronal activity in language areas, independently of the perfusion state. The concurrent SWI venous prominence likely reflects astrocyte-driven hyperemia in adjacent or upstream territories undergoing the excitatory phase. This dissociated pattern underscores the spatially heterogeneous nature of the neurovascular response during FHM2 attacks and challenges a simple ischemia-based interpretation. Astrocytes are integral to neurovascular coupling, actively regulating cerebral blood flow in response to neuronal activity [14]. ATP1A2-related astrocytic dysfunction may therefore result not only in impaired neurotransmitter clearance [13] but also in abnormal cerebrovascular regulation [14, 15]. 

Clinically, acute aphasia with left hemispheric slowing on EEG raised initial concerns for ischemic stroke or focal seizure. However, the absence of diffusion restriction on DWI, the lack of epileptiform discharges, and spontaneous resolution within 24 h supported a diagnosis of hemiplegic migraine—ultimately confirmed by *ATP1A2* mutation [1, 2]. While aphasic aura is less common than visual or sensory manifestations, it is well-documented feature of FHM [2] and represents a critical diagnostic consideration in young patients presenting with acute language disturbance and non-ischemic imaging [12]. 

From a diagnostic standpoint, this case emphasizes the critical importance of recognizing hemiplegic migraine as a potent stroke mimic, particularly when vascular imaging demonstrates reversible or non-territorial abnormalities [6]. Misinterpreting TOF-MRA findings as fixed arterial occlusion or viewing SWI venous prominence as definitive, simple hyperperfusion may lead to inappropriate and potentially harmful interventions, such as intravenous thrombolysis [2, 10]. Therefore, a comprehensive diagnostic strategy-integrating the clinical evolution of symptoms, multimodal imaging findings, and, where familial history is suggestive, genetic testing for mutations such as *ATP1A2*-is essential to prevent overtreatment and ensure accurate management [18]. 

Several limitations of this study should be acknowledged. First, quantitative perfusion imaging techniques, such as arterial spin labeling or dynamic susceptibility contrast MRI, were not performed, precluding direct measurement of regional cerebral blood flow and volume. Second, CTA or CT perfusion was not performed at initial evaluation; as the patient had already exceeded the thrombolysis window and was not a candidate for IV intervention, MRI was prioritized instead. Third, follow-up TOF-MRA could not be obtained due to financial constraints; clinical resolution and SWI normalization at day 3 indirectly support a reversible etiology.

## Conclusion

This case of genetically confirmed FHM2 demonstrates simultaneous reduced macrovascular flow signal on TOF-MRA and cortical venous prominence on SWI, suggestive of dissociated neurovascular dynamics within the same cerebral hemisphere during a migraine attack. This previously undescribed pattern underscores the importance of multimodal neuroimaging and genetic testing in distinguishing FHM2 from acute ischemic stroke and avoiding inappropriate thrombolysis.

## Data Availability

All data generated or analyzed during this study are included in this published article. Further details regarding the clinical and genetic data are available from the corresponding author upon reasonable request.
